# Supplementary material for: A mast cell receptor is associated with fibrosis and itch in systemic sclerosis
Source: bioRxiv. 2026 Oct 9:2026.10.02.756290. Preprint. [Version 1] doi: 10.64898/2026.10.02.756290 (PMC13692034; doi:10.64898/2026.10.02.756290)
Supplement: Supplementary file 1 [file NIHPP2026.10.02.756290V1-supplement-1.pdf]

bioRxiv preprint doi: https://doi.org/10.64898/2026.10.02.756290; this version posted October 10, 2026. The copyright holder for this preprint (which was not certified by peer review) is the author/funder, who has granted bioRxiv a license to display the preprint in perpetuity. It is made available under a CC-BY 4.0 International license.

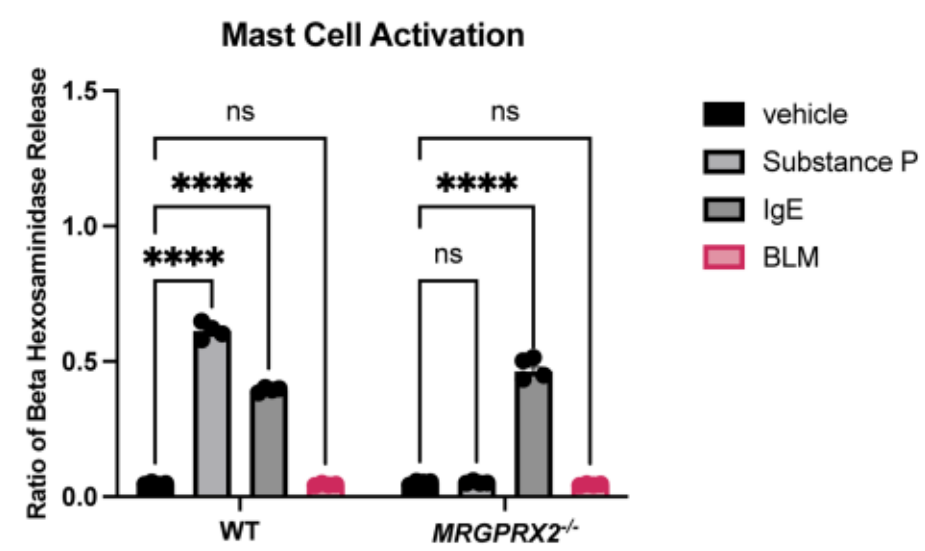

Supplementary Figure 1. BLM does not activate MRGPRX2. WT and *MRGPRX2*$^{-/-}$ LAD2 human mast cell beta-hexosaminidase release after BLM treatment, using SP and IgE as positive controls (*n*=4 biological replicates for all conditions). Statistical analysis: two-way ANOVA with Sidak's multiple comparisons test (A-B). ****$P < 0.0001$.

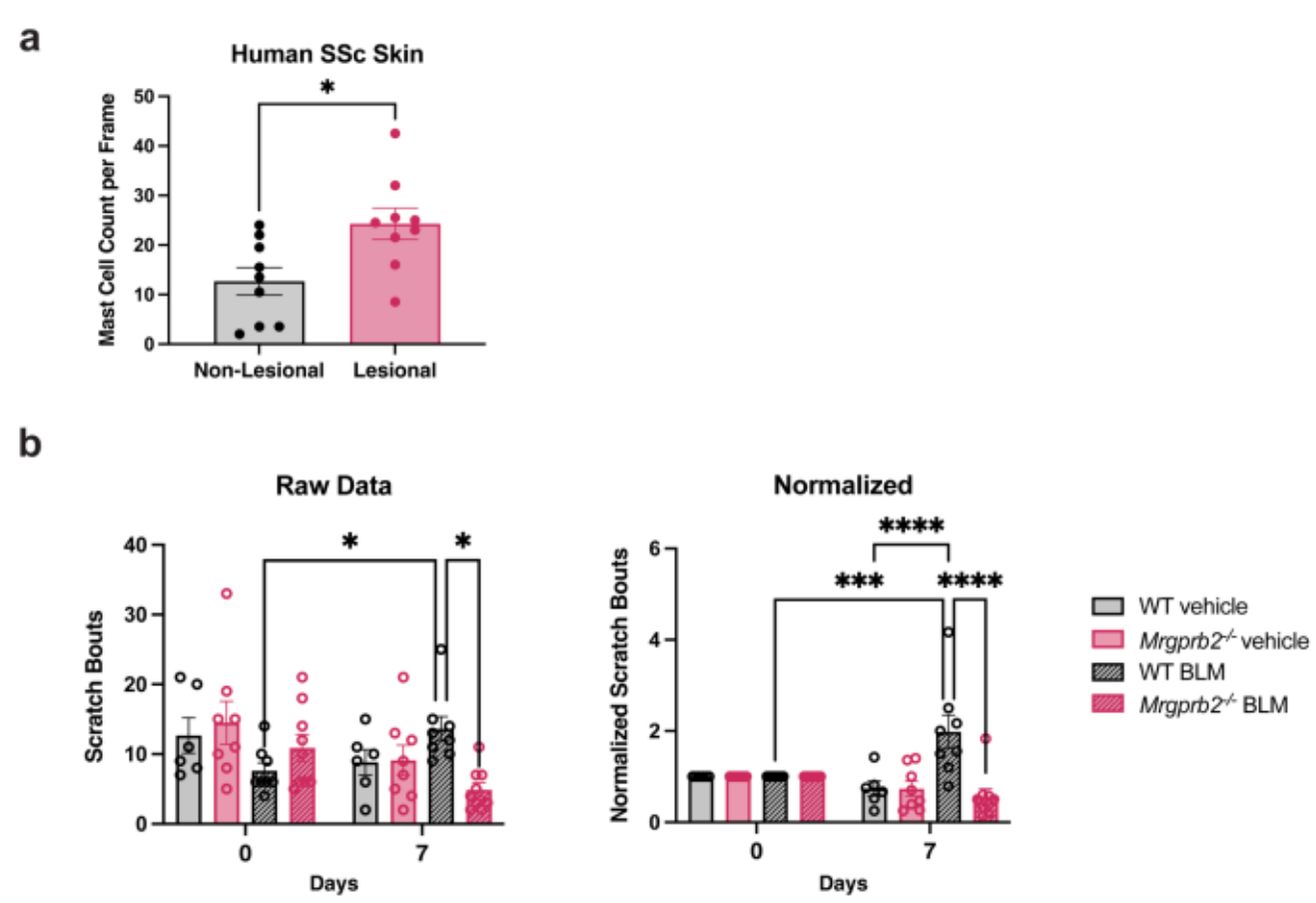

Supplementary Figure 2. Mrgprb2 contributes to itch in the bleomycin model of skin fibrosis. (a) Same data as seen in Figure 2e, plotted as pooled non-lesional and lesional samples with no pairing, and with bar graphs to outline means and SEM. **(b)** Same data as seen in Figure 2g, plotted in bar graph format to outline means and SEM. (left) Raw data and (right) data normalized to baseline itch counts for each mouse. Statistical analysis: two-sided Student's t-test (a), two-way ANOVA with Tukey's multiple comparisons test (b). *$P < 0.05$, ***$P < 0.001$, ****$P < 0.0001$.

bioRxiv preprint doi: https://doi.org/10.64898/2026.10.02.756290; this version posted October 10, 2026. The copyright holder for this preprint (which was not certified by peer review) is the author/funder, who has granted bioRxiv a license to display the preprint in perpetuity. It is made available under a CC-BY 4.0 International license.

Supplementary Table 1. Demographic, Clinical, and Serologic Features of Patients with SSc (n = 11)

Characteristic	n (%)
Demographics	
Age at biopsy, mean (SD), y	51.3 (10.8)
Duration of disease at biopsy, mean (SD), y	1.8 (2.1)
Female sex, n (%)	9 (81.8)
Race	
White race, n (%)	7 (63.6)
Black race, n (%)	2 (18.2)
Ethnicity, n (%)	
Hispanic, n (%)	2 (18.2)
Smoking History	
Current, n (%)	2 (18.2)
Former, n (%)	3 (27.3)
Family history of autoimmune disease, n (%)	4 (36.4)
Comorbidities	
Hypertension, n (%) [a]	7 (63.6)
Dyslipidemia, n (%) [b]	3 (27.3)
COPD, n (%) [a]	2 (18.2)
SSc Disease Features (at time of biopsy)	
Diffuse cutaneous subtype, n (%)	9 (81.8)
Limited cutaneous subtype, n (%)	1 (9.1)
Raynaud phenomenon, n (%)	7 (63.6)
Modified Rodnan skin score, mean (SD) [a]	14.7 (7.2)
Scleroderma renal crisis, n (%)	1 (9.1)
Vascular and Cutaneous Involvement (at time of biopsy)	
Digital ulcers, n (%)	1 (9.1)
Digital pitting, n (%)	2 (18.2)
Telangiectasias, n (%)	5 (45.5)
Calcinosis, n (%)	1 (9.1)
Systemic Involvement	
Dyspnea, n (%) [a]	7 (63.6)
Arthralgia, n (%)	5 (45.5)
Autoantibodies	
ANA, n (%)	9 (81.8)
Anti-centromere, n (%)	1 (9.1)
Anti- Scl-70 (topoisomerase I), n (%)	2 (18.2)
Anti-RNA polymerase III, n (%)	4 (36.4)
Pulmonary Function (closest to time of biopsy)	
FVC, % predicted, mean (SD) [a]	87.7 (16.8)

bioRxiv preprint doi: https://doi.org/10.64898/2026.10.02.756290; this version posted October 10, 2026. The copyright holder for this preprint (which was not certified by peer review) is the author/funder, who has granted bioRxiv a license to display the preprint in perpetuity. It is made available under a CC-BY 4.0 International license.

DLCO, % predicted, mean (SD) [b]	67.1 (17.2)
Pulmonary hypertension, n (%)	1 (9.1)
Treatment	
Any immunomodulatory therapy, n (%)	9 (81.8)
Hydroxychloroquine, n (%)	2 (18.8)
IVIG, n (%)	2 (18.8)
Methotrexate, n (%)	1 (9.1)
Mycophenolate Mofetil, n (%)	6 (54.5)
Prednisone, n (%)	1 (9.1)
Rituximab, n (%)	1 (9.1)

Abbreviations: COPD, chronic obstructive pulmonary disease; ANA, antinuclear antibody; FVC, forced vital capacity; DLCO, diffusing capacity of the lungs for carbon monoxide; IVIG, intravenous immunoglobulin. Duration of disease at biopsy is from the time of disease diagnosis per chart review. Treatments indicates use of these drugs at any point for SSc up until time of biopsy.
[a] Data missing for 1 patient.
[b] Data missing for 2 patients.
Percentages are calculated among patients with non-missing data. Continuous variables are shown as mean (SD); categorical variables as n (%).

bioRxiv preprint doi: https://doi.org/10.64898/2026.10.02.756290; this version posted October 10, 2026. The copyright holder for this preprint (which was not certified by peer review) is the author/funder, who has granted bioRxiv a license to display the preprint in perpetuity. It is made available under a CC-BY 4.0 International license.

Supplementary Table 2. Regression of Skin Severity Outcomes and Lesional Mast Cell Counts (n = 11)

Model	Pearson or Spearman Coefficient	95% CI	P value	n
Modified Rodnan skin score – Pearson correlation				
Unadjusted	r = 0.63	[-0.00, +0.90]	0.052	10
+ Age			**0.025**	10
+ Sex			**0.036**	10
+ Race			0.239	10
+ Ethnicity			**0.004**	10
+ Subtype			0.070	10
+ Smoking			0.058	10
Patient-reported skin tightness (0–4) – Spearman rank correlation				
Unadjusted	ρ = 0.756	[+0.11, +0.95]	**0.030**	8
+ Age			**0.038**	8
+ Sex			-	8
+ Race			**0.016**	8
+ Ethnicity			**0.025**	8
+ Subtype			0.097	7
+ Smoking			**0.035**	8
Patient-reported skin hardness (0–4) – Spearman rank correlation				
Unadjusted	ρ = 0.718	[-0.45, +0.98]	0.172	5
+ Age			0.272	5
+ Sex			-	5
+ Race			0.287	5
+ Ethnicity			0.287	5
+ Subtype			0.144	5
+ Smoking			0.109	5

Each outcome was regressed unadjusted on lesional mast cell count, then adjusted for a single covariate at a time (age at biopsy, sex, race, ethnicity, disease subtype, and smoking history). Pearson and Spearman coefficients and 95% confidence intervals are shown for the unadjusted model; adjusted rows report the covariate-adjusted p value. Bold p values are < .05. "-" indicates the covariate was not evaluated in that subset (all patients with patient-reported skin outcomes are female). mRSS is continuous (linear regression; with Pearson r). Skin tightness and hardness are ordinal items scored 0-4 (Spearman rank correlation unadjusted, partial rank regression adjusted).
